# General Criminal Dynamic Risk and Strength Factors Predict Short-Term General Recidivism Outcomes Among People Convicted of Sexual Crime During Community Supervision

**DOI:** 10.1177/10790632221146499

**Published:** 2022-12-17

**Authors:** Melissa S. de Roos, Caleb D. Lloyd, Ralph C. Serin

**Affiliations:** 1 Department of Psychology, Education and Child Studies, 6984 Erasmus University Rotterdam, Rotterdam, Netherlands; 2 Centre for Forensic Behavioural Science, 3783 Swinburne University of Technology and Forensicare, Alphington, VIC, Australia; 3 Department of Psychology, 414990 Carleton University, Ottawa, ON, Canada

**Keywords:** dynamic risk assessment for offender re-entry, acute dynamic risk, reassessment, parole, recidivism

## Abstract

There are clinical practice and operational reasons why it may be appropriate to primarily focus on general risk factors when supervising people convicted of sexual crime in the community. General risk domains may be particularly relevant when supervision officers engage in frequent reassessment of acute dynamic risk factors. We tested the ability of a case management tool, the Dynamic Risk Assessment for Offender Re-entry, to discriminate community based, short-term general (all outcome) recidivism versus nonrecidivism among people convicted of sexual crime (*n* = 562). We tested the predictive discrimination validity of each DRAOR item and then subscale scores in univariate and multivariate models (also controlling for general static risk). DRAOR scores were associated with general recidivism outcomes and effect sizes were generally similar or stronger compared to models with people convicted of nonsexual crime (*n* = 2854). DRAOR Acute scores were consistently and incrementally related to general recidivism outcomes beyond other scores. In practice, case managers should remain aware that people convicted of sexual crime are at risk for nonsexual recidivism outcomes and assess problematic functioning broadly alongside problems in sexual domains. Clinically, interconnection among domains potentially provides multiple avenues for effective intervention.

## Introduction

People convicted of sexual crime represent a relatively small proportion of the correctional population, but the serious interpersonal violation of sexual offending generates strong correctional effort toward reducing sexual recidivism. For example, in New Zealand (the context of the present research), 4.6% of people supervised in the community were convicted of a sexual crime (New Zealand Department of Corrections, 2020). Yet, because sentences for sexual crimes are, on average, twice the length of nonsexual crimes (i.e., eight vs. 4 years; New Zealand Department of Corrections, 2015), people convicted of sexual crime represent a higher relative proportion of people on parole (18.6%; New Zealand Department of Corrections, 2020).

Internationally, several community management strategies (e.g., sex offender registration, community notification, indeterminate civil commitment) are substantially long-term, often applied equally among people with different degrees of risk for recidivism, and have limited, unknown, and/or unlikely efficacy for reducing sexual recidivism (Logan, 2020). An alternate evidence-supported approach involves assessing long-term risk, applying management resources only among those who are relatively higher risk (Hanson et al., 2009; 2012), adjusting estimated levels of risk as these decline with age and time recidivism-free in the community (Hanson et al., 2018; Thornton et al., 2021), and, among those currently at-risk for recidivism, targeting presently active dynamic risk factors (i.e., changeable life domains empirically related to recidivism; Wilson et al., 2020).

This approach is consistent with the risk-need-responsivity (RNR) model (Bonta & Andrews, 2017) that tasks corrections agencies to use risk assessment to allocate resources to those with greatest risk for recidivism, then use evidence-based, client-responsive interventions to address dynamic risk factors. The RNR model identifies antisocial personality features (i.e., impulsivity, negative emotionality), antisocial and/or poor prosocial relationships, poor self-regulation or lifestyle instability, and disregard for legal and social rules as dynamic risk factors.

There are a variety of risk tools of dynamic risk factors designed for the population of people convicted of sexual crimes. A meta-analysis of results from 23 samples representing 14 dynamic tools showed associations between risk scores and future sexual recidivism (Cohen’s *d* = .71) and any recidivism (Cohen’s *d* = .64) with medium-to-large effects (van den Berg et al., 2018). Using dynamic risk scores alone, van den Berg et al. (2018) meta-analysis showed prediction effects commensurate to effects when using static risk scores alone (e.g., Hanson & Morton-Bourgon, 2009); however, when applying incremental models using 13 samples, increases in prediction over static scores suggested the effect size associated with dynamic scores was small. Thus, in the RNR model, assessment of dynamic risk factors is critical for defining the focus of intervention; but, for prediction purposes, practice models must account for the high overlap in prediction power shared by static and dynamic scores, for example, by using criminal history to screen for risk level then assessing dynamic factors only among those eligible for intervention.

Dynamic assessments in van den Berg et al. (2018) analyses were mostly “static” in that there was only one assessment of theoretically dynamic factors. This is consistent with Douglas and Skeem’s (2005) earlier critique that empirically establishing the properties of dynamic factors has lagged behind their proposed theoretical function. Combining the six available samples using a reassessment methodology (i.e., initial and subsequent scores), van den Berg et al. (2018) calculated a weighted average hazard ratio for reassessment predicting sexual recidivism (*HR* = 0.91). Most results were from four samples assessing pre- and post-program Violence Risk Scale-Sex Offense (VRS-SO; Wong et al., 2003 - 2017) scores, with both scores recorded prior to prison release and before people were at risk in the community (see Klepfisz et al., 2022, for methodological critiques).

By contrast, more recently emerging evidence shows community-based reassessments of dynamic risk factors indicate current risk for general and sexual recidivism better than prior assessments even as recent as at release (Babchishin & Hanson, 2020; Hanson et al., 2021). This suggests there are meaningful fluctuations in risk factors and associated risk for recidivism within the community and, as such, corrections agencies should in some way consider reassessed dynamic risk information to determine management decisions in this context.

By implementing ongoing reassessment strategies, agencies may benefit from increased staff attention and intervention toward dynamic risk factors. Yet, benefits are not guaranteed. Related costs include staff time, policy shifts, training, quality assurance mechanisms, and organizational buy-in. Costs may be further tied to whether corrections agencies assign some supervision officers to caseloads populated only with people convicted of sexual crimes versus create a whole-system training approach, using a single tool to assess risk for general recidivism and/or one common set of dynamic risk factors. For example, when New Zealand implemented a structured case management tool designed for the general population (Dynamic Risk Assessment for Offender Re-entry, or DRAOR; Serin, 2007) within its parole services, staff rated all clients using DRAOR, including those with convictions for sexual crime.

This implementation strategy created an opportunity for us to examine whether DRAOR scores predict general recidivism among people on parole for convictions for sexual crime. Because reassessments occurred relatively frequently (weekly to fortnightly), we examined the relationship between risk scores and short-term, imminent recidivism (prior to the next assessment). However, the total follow-up timeframe (1 year) was too short to observe a meaningful base rate of sexual recidivism, so our validation focused on whether DRAOR scores discriminate imminent general (any outcome) recidivism versus non-recidivism.

### Nonsexual Risk Factors Predict General Recidivism in Samples with Sexual Crimes

People convicted of sexual crime are more likely to recidivate with nonsexual than sexual recidivism (Hanson & Morton-Bourgon, 2009). Criminality in this population is more generalized than specialized, including within subgroups differentiated by either child or adult victims; however, patterns of specialization in sexual crime are somewhat more evident among people with child victims (Lussier et al., 2005), suggesting sex-specific risk factors may be more relevant for this subgroup.

Still, if a corrections agency wants to address general recidivism risk among people convicted of sexual crimes or wants to standardize core assessment procedures, they can rely on general risk tools that predict general recidivism in all sub-populations, including people convicted of sexual crime. Examples include the Level of Service (LS) family of tools, i.e., Level of Service Inventory-Revised (LSI-R; Andrews & Bonta, 1995), Level of Service/Case Management Inventory (LS/CMI; Andrews et al., 2004), and Youth Level of Service/Case Management Inventory (YLS/CMI; Hoge et al., 2002), or Post Conviction Risk Assessment (PCRA; Johnson et al., 2011). Among adults convicted of sexual crime, LSI-R and LS/CMI scores showed large effects for general recidivism prediction (AUCs = .69 to .77; Ragusa-Salerno et al., 2013; Tsao & Chu, 2021; Wormith et al., 2012). Effects were similarly large using PCRA scores (Harrell’s *c* = .73 for any arrest, *c* = .77 for any revocation; Cohen, 2018). Predicting general recidivism among adult women convicted of sexual crimes showed a medium effect for LSI-R scores (*c* = .65; Marshall et al., 2022). Similarly, prediction generally showed medium effects among youth with sexual crimes using YLS/CMI scores, but with more inconsistency and greater range due to most samples being small (AUC = .58 to .73; Caldwell & Dickinson, 2009; Chu et al., 2012; Morton, 2003; Schmidt et al., 2016; Viljoen et al., 2009).

### Community Case Management and Nonsexual Risk Factors for Predicting Imminence

The LS family of tools and PCRA are more comprehensive, longer-term dynamic risk tools, whereas, in the context of short-term imminent risk in the community, there has not yet been similar tests of the relationship between general risk tools and general recidivism among people convicted of sexual crime. However, Acute-2007 (Hanson et al., 2007) assesses many more general (*rejection of supervision, hostility, change in social supports, emotional collapse, substance use, and victim access*) rather than sex-specific risk factors (*sexual preoccupation*) among its list of faster changing acute dynamic risk factors. This balance toward general risk factors is consistent with other sexual recidivism risk tools. Still, scoring context and manual guidance may be critical. For example, it may be meaningfully different for prediction if raters score the simple presence of *substance use* on a general risk tool, but only consider current *substance use* to be problematic if similar use previously occurred in the context of prior sexual offending. Similarly, general *hostility* may not have the same predictive effects as the more specific consideration of *hostility toward women* among people convicted of sexual crime. Or, it may matter if raters consider only potential child victims when scoring *victim access* rather than considering potential victims of family violence, stalking, fraud, etc. Despite broad conceptual similarities across tools, predictive effects may depend on definition specificity as it applies to the specific sub-population or may depend on concordance between items and outcome behavior.

Still, Acute-2007 assesses general risk well enough for scores to be related to general recidivism and, importantly, Babchishin and Hanson (2020) showed that dynamically updated Acute-2007 scores across an average 7.2-year follow-up were more strongly related to subsequent sexual and general recidivism relative to scores recorded at the beginning of community supervision. Similarly, Hanson et al. (2021) reported that updated Sex Offender Treatment Intervention and Progress Scale (SOTIPS; McGrath et al., 2013) scores over an average 3-year follow-up incrementally discriminated sexual and general recidivism beyond static risk scores. Designed mostly for 6-month reassessment, items on SOTIPS share some similarity with the longer-term dynamic factors of Stable-2007 (Hanson et al., 2007) and similarly include relatively more general than sexual risk items (i.e., 5 out of 16 SOTIPS items are sex-specific factors). Thus, counts of mostly general problem lifestyle factors assessed in the community can signal current risk of diverse recidivism outcomes among people convicted of sexual crime.

Two DRAOR subscales (Stable and Acute) generally demonstrate conceptual concordance with SOTIPS and Acute-2007, respectively, by assessing similar general risk factors yet omitting all sex-specific factors. Several research teams have validated the predictive discrimination of DRAOR scores in New Zealand, including within the general population of people on parole (Lloyd et al., 2020), high-risk men on parole (Davies et al., 2022; Yesberg & Polaschek, 2015), and females either on parole or community-based sentences (Scanlan et al., 2020; Yesberg et al., 2015). Further, DRAOR risk domain scores decline over the first 12 months of reassessments following release from incarceration whereas Protect domain scores increase (Lloyd et al., 2020; Polaschek & Yesberg, 2018) and, compared to scores assessed at the time of release, reassessed scores better discriminate recidivism from non-recidivism (Davies et al., 2022; Lloyd et al., 2020; Stone et al., 2021).

However, there are limited evaluations of DRAOR for assessing risk for recidivism among people convicted of sexual crimes and neither used reassessment to test imminent prediction. In one unpublished thesis, Smeth (2013) reported that each DRAOR subscale (assessed at one point in time) discriminated future parole violations after controlling for Static-99 scores among 203 men convicted of sexual crime in the United States, but DRAOR subscales did not discriminate sexual recidivism over the maximum 8.7-year follow-up. In another unpublished thesis, Averill (2016) reported univariate analyses in which each DRAOR subscale (assessed once) discriminated sexual and any recidivism outcomes among 850 men sentenced for sexual offending in New Zealand across a maximum 4.8-year follow-up. Results were less consistent across a variety of multivariate models, but each independent DRAOR subscale incrementally discriminated general recidivism outcomes after controlling for general static risk whereas none of the three DRAOR subscales incrementally discriminated sexual recidivism outcomes after controlling for static risk for sexual recidivism.

### The Current Study

Our evaluation of DRAOR among people convicted of sexual crimes had several aims. First, following research on the LS family of tools and PCRA, and using stronger analytic techniques than prior evaluations using DRAOR with samples of people convicted of sexual crime, we examined whether each individual general risk and strength item on DRAOR discriminated general recidivism. Item-level analysis is relevant because, although many DRAOR scale items are similar to items on other tools, it may be critical that the DRAOR manual is not specifically designed for people convicted of sexual crimes. We repeated models to explore all recidivism outcomes (including breaches of parole) and only criminal recidivism outcomes (excluding breaches because breaches may be less serious and more foreseen by supervision officers recording DRAOR scores). Second, following several findings (Ragusa-Salerno et al., 2013; Schmidt et al., 2016; Wormith et al., 2012) that prediction effect sizes for LS tools were stronger for predicting general recidivism among people with sexual crimes compared to people without sexual convictions, we similarly examined both groups to compare strength of effects and to see if these prior findings may replicate using DRAOR.

Third, following van den Berg et al. (2018) meta-analytic summary of weaker incremental prediction among dynamic risk scores after first accounting for static risk scores, we used DRAOR scale scores in both independent and incremental models. Finally, following (Lussier et al., 2015) finding of greater criminal specificity among people with convictions of sexual crimes with child victims, we tested our models across subgroups of people with either child versus adult victims of sexual crime (based on their current sexual conviction).

## Method

### Participants

New Zealand Department of Corrections provided a dataset containing information about all people supervised on parole over a 2-year period (*N* = 3694; April 2010 to March 2012). After excluding those with missing risk assessments (DRAOR or static risk scores), the final dataset represented 92.5% of the parole population in New Zealand during this time (*N* = 3416). Prior research using this dataset concluded that DRAOR scores (1) demonstrate measurement invariance across time (Lloyd, Hanson, et al., 2020), (2) were associated with future general and violent recidivism within the whole sample (Coulter et al., 2022; Lloyd, Hanson, et al., 2020; Stone et al., 2021), (3) were more strongly related to general and violent recidivism when using updated scores compared to initial or averaged scores (Lloyd, Hanson, et al., 2020; Stone et al., 2021; Stone et al., 2022; Stone et al., 2023), and (4) are best conceptualized as assessing risk and promotive factors (Lloyd, Perley-Robertson, & Serin, 2020).

We divided the sample into two subgroups based on whether parole supervision resulted from a conviction for a sexual or a nonsexual offense. The subsample of people convicted for sexual crime partially overlaps with people in the dataset generated by a different research team and analyzed by Averill (2016). We display the demographic characteristics of the two groups in our dataset in Table 1. We were unable to determine whether people with a nonsexual index offense may have had a criminal history that included any prior sexual offenses. Among people with a sexual index offense, identified victims were more often under the age of 16 (61.2%) than aged 16 or older (i.e., the age of consent in New Zealand; 34.0%). Victim age was unspecified for 27 (4.8%) of the 562 people with a sexual index offense.

**Table 1.** Descriptive Statistics and Mean Risk Static Risk and DRAOR Subscale Scores for People with a Sexual and Nonsexual Index Offense at the Time of Release from Incarceration, with Chi-Square and Analysis of Variance (ANOVA) Comparing Frequencies and Means.

Statistic	Sexual Index Offense (*n* = 562)			Nonsexual Index Offense (*n* = 2854)	Group Comparison and Effect Sizes
	Full Subsample	Victim Under 16 Years (*n* = 344)	Victim 16 Years or Older (*n* = 191)		
	*n* (%)	*n* (%)	*n* (%)	*n* (%)	*χ*^*2*^ (*df*); *p*; Cramer’s *V*
Male	561 (99.8)	343 (99.7)	191 (100.0)	2610 (91.5)	
Ethnicity					76.97 (8); *p* < .001; .11
New Zealand European	254 (45.2)	179 (52.0)	58 (30.4)	895 (31.3)	
New Zealand Māori	211 (37.5)	106 (30.8)	99 (51.8)	1481 (51.9)	
Pacific Islander	58 (10.3)	32 (9.3)	24 (12.6)	282 (9.9)	
Other European	29 (5.2)	22 (6.4)	5 (2.6)	108 (3.8)	
Other ethnicity	10 (1.8)	5 (1.5)	5 (2.6)	88 (3.1)	
Marital status					161.9 (8); *p* < .001; .16
Never married	251 (44.7)	131 (38.1)	108 (56.6)	1987 (69.6)	
Married (Legal or De facto)	135 (24.0)	85 (24.7)	39 (20.4)	430 (15.1)	
Divorced or Separated	120 (21.3)	86 (25.0)	31 (16.2)	262 (9.2)	
Widowed	6 (1.1)	6 (1.7)	0 (0)	16 (0.5)	
Other/Unknown	50 (8.9)	36 (10.5)	13 (6.8)	159 (5.6)	

	*M* (*SD*)	*M* (*SD*)	*M* (*SD*)	*M* (*SD*)	*F* (*df*); *p*; *η*^*2*^ [95% CI]
Age (at release)	44.0 (14.1)	46.1^a^ (15.1)	39.9^b^ (11.2)	33.3^c^ (10.2)	231.6 (2); *p* < .001; .12 [.10, .14]
Risk score (at release)					
RoC*RoI (static risk)	.31 (.25)	.26^a^ (.23)	.42^b^ (.25)	.54^c^ (.23)	239.1 (2); *p* < .001; .12 [.10, .14]
DRAOR stable	6.19 (2.61)	6.10 (2.58)	6.43 (2.66)	6.35 (2.51)	10.6 (2); *p* = .19; .001 [.000, .004]
DRAOR acute	5.83 (2.29)	5.69 (2.19)	6.06 (2.46)	5.91 (2.43)	9.70 (2); *p* = .19; .001 [.000, .004]
DRAOR protect	6.12 (2.45)	6.19 (2.43)	6.07 (2.45)	6.07 (2.38)	2.29 (2); *p* = .67; .001 [.000, .002]

*Note*. RoC*RoI = risk of reconviction*risk of re-imprisonment (Bakker et al., 1999); DRAOR = dynamic risk assessment for offender re-entry (Serin, 2007).

Different superscripts indicate groups with statistically significant differences.

For each group, Table 1 displays participant gender, ethnicity, marital status, and age. Compared to people convicted of a sexual offense, people with a nonsexual index offense were more likely to be younger, never married, and identify as New Zealand Māori. All but one of the 245 female participants were convicted for nonsexual index offenses. Compared to people with known victims under the age of 16, the demographic characteristics (ethnicity, marital status, and age) among people convicted of a sexual offense with older victims were more similar to the demographic characteristics of people with a nonsexual index offense.

### Measures

#### Recidivism

New Zealand Department of Corrections provided a database of official record recidivism events. We used two definitions of recidivism: (1) any recidivism, including either the first violation of conditions of supervision or the first new criminal charge after release from incarceration, and (2) criminal recidivism, excluding violations of conditions of supervision and including only new criminal charges. Because we restricted our analyses to the first 12 months following release from incarceration, the mean follow-up time among people convicted of a nonsexual index offense was 17.5 weeks (*SD* = 13.6, median = 15 weeks) and was 19.2 weeks (*SD* = 14.0, median = 17 weeks) among people convicted of a sexual index offense.

#### Static Risk

Risk of Reconviction*Risk of Re-imprisonment (RoC*RoI; Bakker et al., 1999) is New Zealand Department of Corrections’ internally developed 16-item static risk assessment tool, measuring demographic (gender and current age) and criminal history (indicators of criminal career, crime frequency, and crime severity) variables. RoC*RoI scores are normed to indicate the likelihood of a return to incarceration within 5 years among the population of people convicted of a crime in New Zealand. Scores discriminate future recidivism from non-recidivism (AUC = .76; Bakker et al., 1999; Nadesu, 2007). At entry into and exit from incarceration, a computer automatically scores RoC*RoI using item information stored in the department’s computer systems based on the logistic model described by Bakker et al. (1999). The algorithm produces a score between 0 and 1 that represents a likelihood; for example, a RoC*RoI score of .63 represents a 63% likelihood of return to incarceration within 5 years.

#### Dynamic Risk and Strength Factors

*Dynamic Risk Assessment for Offender Re-entry* (DRAOR; Serin, 2007) is a 19-item case management tool designed to guide how supervision officers conceptualize case management needs and make management decisions about clients on community supervision. DRAOR items are organized into Stable dynamic risk items (antisocial attitudes, traits, and associations), Acute dynamic risk items (life circumstances and destabilizing factors), and Protect dynamic strength items (prosocial self-perceptions and prosocial connectedness). We display all DRAOR items in Table 2. Raters score the six DRAOR Stable and seven DRAOR Acute items as *not a problem* (0), *slight or possible problem* (1), or *definite problem* (2), for possible scores between 0–12 and 0–14, respectively, and the six DRAOR Protect items as *not an asset* (0), *slight or possible asset* (1), or *definite asset* (2), for possible scores between 0–12. As a field-implemented tool, there is no information on interrater reliability in this sample. However, training to score DRAOR was centralized and standardized for all staff within New Zealand Department of Corrections, with consistent oversight from staff supervisors to address quality assurance.

**Table 2.** Concordance Indices and 95% Confidence Intervals Derived from Cox Regression with Time-Varying Predictor Models Predicting Any Recidivism Outcome (Including Breaches of Parole) Using DRAOR Item Scores.

DRAOR Item	Sexual Index Offense(*n* = 597)	Nonsexual Index Offense(*n* = 3051)
	*c*-index [95% CI]	*c*-index [95% CI]
DRAOR stable subscale		
Antisocial peer associations	0.70 [0.66, 0.73]	0.59 [0.58, 0.61]
Negative attitudes to authority	0.63 [0.59, 0.67]	0.60 [0.58, 0.61]
Problems with Impulse control	0.65 [0.60, 0.69]	0.61 [0.60, 0.63]
Antisocial problem-solving	0.62 [0.57, 0.66]	0.60 [0.58, 0.61]
Sense of entitlement	0.60 [0.56, 0.64]	0.58 [0.57, 0.60]
Poor attachment to others	0.57 [0.53, 0.62]	0.57 [0.56, 0.58]
DRAOR acute subscale		
Problems with substance misuse	0.63 [0.59, 0.68]	0.58 [0.57, 0.60]
Anger/Hostility	0.56 [0.52, 0.61]	0.57 [0.55, 0.58]
Access to victims	0.59 [0.54, 0.63]	0.56 [0.55, 0.58]
Negative mood	0.56 [0.52, 0.61]	0.56 [0.54, 0.57]
Problems with employment	0.61 [0.56, 0.65]	0.58 [0.56, 0.59]
Interpersonal relationship problems	0.56 [0.52, 0.60]	0.57 [0.56, 0.58]
Problems with living situation	0.61 [0.56, 0.66]	0.61 [0.59, 0.62]
DRAOR protect subscale		
Responsive to prosocial advice	0.60 [0.56, 0.64]	0.59 [0.57, 0.60]
Prosocial identity	0.61 [0.57, 0.66]	0.61 [0.60, 0.62]
High & realistic expectations	0.62 [0.57, 0.66]	0.58 [0.57, 0.60]
Prosocial cost/Benefit ratio	0.60 [0.55, 0.64]	0.60 [0.58, 0.61]
Prosocial social support	0.60 [0.56, 0.64]	0.59 [0.58, 0.61]
Prosocial social control	0.62 [0.57, 0.66]	0.59 [0.58, 0.61]

*Note*. Sexual Index *n* = 562 people, *n* = 597 release events, *n* = 17,175 weeks of follow-up, *n* recidivismevents = 132. Nonsexual Index *n* = 2854 people, *n* = 3051 release events, *n* = 74,770 weeks of follow-up, *n* recidivism events = 1210.

DRAOR = dynamic risk assessment for offender re-entry (Serin, 2007); *c*-index = concordance index;CI = confidence interval.

All values *p* ≤ .01.

#### Demographic Information

New Zealand Department of Corrections provided a database with participants’ date of birth, gender, ethnicity, marital status, and index offense. Index offense categories indicated whether the victim was under the age of 16 or older, except for 27 sexual index offenses that did not specify victim age.

### Procedure

We obtained ethics approval for our use of the data from institutional review boards at the following institutions: Carleton University (#11-113) and Swinburne University of Technology (#2017/230). In New Zealand, supervision officers score all 19 DRAOR items for each client at every reporting session or “quality contact” (i.e., any communication long enough to reassess the items). Policy dictates supervision officers fully reconsider each DRAOR Acute item at reassessment but keep a “watching brief” on Stable and Protect items. For each client, the rate of reassessment depends upon the frequency of contact (determined by static risk and the amount of time since release from incarceration); this typically varies between twice weekly to fortnightly.

During the time period recorded in the dataset, some people experienced re-incarceration and subsequent re-release. Because predictor information updated with each new release, we retained each release event for analysis (see Howard & Dixon, 2013; Lloyd et al., 2020), resulting in 3648 release events (*n* = 597 release events among 562 people with sexual index offenses and *n* = 3051 release events among 2854 people with nonsexual index offenses). Across all participants, we observed 91,945 weeks of follow-up time in the community.

For most of the sample, parole supervision orders and DRAOR assessments ceased within 12 months following release from incarceration; records of DRAOR scores extended beyond 12 months for only 16.5% of the sample. To maintain consistent interpretation of the outcome (i.e., imminent recidivism, or recidivism within 1–2 weeks following the most recent DRAOR assessment), we truncated the follow-up at 12 months. We organized all assessments to be prospective predictors of subsequent recidivism, removing any assessments that occurred on the same day as a recidivism event to avoid the possibility that supervision officers recorded scores retrospectively after a recidivism outcome.

### Plan of Analysis

For both groups of people supervised on parole (those convicted of sexual and nonsexual offenses) and for each of the three DRAOR subscale domains (that contain theoretically distinct information), we calculated descriptive information (mean subscale scores and standard deviations) recorded at the first assessment at the time of release from incarceration. For 28.9%, this assessment occurred on the date of release; the remaining occurred within days or up to 4 weeks shortly prior to release or within the first days up to 4 weeks after release. We also calculated mean static risk scores (RoC*RoI) and demographic statistics. Further, we calculated descriptive information for two subgroups of people convicted for sexual offenses, i.e., people with identified victims under the age of 16 and people with victims 16 years and older. We used chi-square and analysis of variance (ANOVA) analyses to compare differences in demographics and assessment scores across people with (1) nonsexual offenses, (2) sexual offenses with identified victims under the age of 16, and (3) sexual offenses with victims 16 years and older.

Next, to evaluate whether each of the 19 DRAOR items was associated with recidivism outcome (defined broadly to include breaches of conditions of supervision orders) among people convicted for sexual offenses, we estimated Cox regression survival analysis models, entering each item score as the predictor and recidivism as the outcome. We estimated 19 models using all assessments within the follow-up period as a predictor that varied with time. This analytic approach retains the temporal structure of all assessments and outcomes for both people with and without recidivism (with release from incarceration as the comparable start point), so, at any one time, our models compared DRAOR scores among people with current recidivism to DRAOR assessments recorded at the same time for people who had not (yet) recidivated. In these time-varying models, the models use each DRAOR assessment as the predictor until it is superseded and replaced in time by a subsequent assessment. For comparison, we repeated these models with the subsample of people convicted for nonsexual offenses.

In Cox regression, hazard ratio quantifies the difference in recidivism rate associated with each 1-point difference on the scale. Lower Akaike information criterion (AIC) and Bayesian information criterion (BIC) values indicate better fitting models, with a 10-point difference considered strong support for the better-fitting model (Raftery, 1995). We report model concordance (or, the *c*-index; Harrell et al., 1982), a time-specific area under the curve (AUC) value readers can interpret as an AUC statistic that accounts for follow-up time. It summarizes the degree to which the predictor variable discriminates recidivism from non-recidivism, controlling for follow-up time by comparing people only at equivalent points, i.e., the same length of time since release from incarceration.

Next, among each subgroup and definition of recidivism, we estimated Cox regression models by entering (1) DRAOR Stable subscale scores, (2) DRAOR Acute scores, (3) DRAOR Protect scores, and then a multi-predictor model with (4) RoC*RoI static risk scores and all three DRAOR subscale scores. These were also time-varying models. Finally, to ensure that results did not differ by age of identified victim, we repeated the multi-predictor models on the subsamples of people convicted for sexual offenses against a person under the age of 16 versus against a person aged 16 and older. In tables displaying these 17 Cox regression models, we supplement the *c*-index with an *R*^*2*^-analogue (Xu & O’Quigley, 1999) as an additional effect size measure, designated in the table text as *R*^*2*^ (XO). Readers can interpret this statistic as the proportion of variance in the timing of recidivism that is explained by the prediction model. We conducted analyses using *R* version 3 (R Core Team, 2017) using survival (Therneau, 2022), survAUC (Potapov et al., 2012), and risksetROC (Heagerty et al., 2012) packages.

## Results

### Descriptive Statistics and Recidivism Rates

For each subgroup, Table 1 displays mean risk scale scores rated at the time of release from incarceration. Compared to people with nonsexual convictions, static risk scores were lower among people with convictions for sexual offenses. In particular, the average static risk score among people convicted of sexual offenses with victims under the age of 16 (i.e., a RoC*RoI score of .26) estimated a 5-year recidivism rate approximately half the absolute rate of people convicted for nonsexual offenses (e.g., 26% vs. 54%). Among people convicted of sexual offenses with victims aged 16 or older, static risk scores were relatively higher than other people with sexual convictions, but lower than static risk scores of people convicted of nonsexual offenses. By contrast, dynamic risk scores as measured on DRAOR at time of release were not different across the three subgroups to a statistically significant degree. During the follow-up, there was an average of 19.1 (*SD* = 12.1) DRAOR assessments per person.

During the maximum of 1-year follow-up while under community supervision, the subsample of people convicted of nonsexual offenses had an official total recidivism rate (i.e., including breaches) of 39.7%. Excluding breaches and including only criminal recidivism, the recidivism rate was 15.3%. Comparatively, recidivism was relatively rarer among people convicted of sexual offenses, in terms of the total recidivism rate observed for the full subsample (22.1%), people with victims under the age of 16 (18.8%), or people with victims aged 16 or older (29.0%). Including only criminal events, 6.4% of people released from incarceration for a sexual offense recidivated and only six of these recidivism events were for sexual recidivism.

### Predictive Discrimination Validity of Each Dynamic Item and DRAOR Subscale

To explore whether each item on DRAOR discriminated recidivism from non-recidivism within both subsamples (i.e., those convicted of sexual vs. nonsexual offenses), we conducted 38 prediction models using Cox regression survival analysis. In Table 2, we display concordance indices as a model effect size for each model. Results showed that each independent DRAOR item discriminated recidivism for both subgroups of people (i.e., those convicted of sexual vs. nonsexual offenses). Because propensity for recidivism is multidimensional, it is always more useful to combine risk items into aggregate scores. As such, the core goal of these models was to confirm that each of the 19 DRAOR items is relevant for aggregation for prediction for both subsamples. Using Rice and Harris’ (2005) guidelines defining AUC scores as small (.56), medium (.64), and large (.71), effect sizes mainly ranged from small to moderate between *c*-index = .54 to .62. With two exceptions (Anger/Hostility and Interpersonal Relationship Problems on DRAOR Acute), DRAOR items generally discriminated any recidivism outcome equally or slightly stronger among the subsample of people convicted of sexual offenses compared to people convicted with nonsexual offenses.

After combining DRAOR items into scales (Stable, Acute, and Protect) to enter into Cox regression models, each scale discriminated future recidivism from non-recidivism in both subsamples (see Table 3). Effect sizes ranged from medium to large, between *c*-index = .65 to .78. In each case (whether using any or criminal recidivism as the outcome), the magnitude of the predictive discrimination effect was slightly stronger in the subsample of people convicted of sexual offenses. The predictive discrimination effect was stronger when removing breaches of supervision from the definition of recidivism and examining criminal recidivism events only.

**Table 3.** Cox Regression with Time-Varying Predictor Models Predicting Time to Recidivism Among People Incarcerated for a Sexual or Nonsexual Offense Using Static Risk (RoC*RoI) and DRAOR Subscale Scores.

Parameter	All Recidivism Outcomes (Including Breaches of Parole)				Criminal Recidivism Outcomes			
	Sexual Index Offense(*n* = 597)		Nonsexual Index Offense(*n* = 3051)		Sexual Index Offense(*n* = 597)		Nonsexual Index Offense(*n* = 3051)	
	*B* (*SE*)	HR [95% CI]	*B* (*SE*)	HR [95% CI]	*B* (*SE*)	HR [95% CI]	*B* (*SE*)	HR [95% CI]
DRAOR stable	0.23*** (0.031)	1.25 [1.18, 1.33]	0.17*** (0.0101)	1.19 [1.17, 1.21]	0.296*** (0.058)	1.34 [1.20, 1.51]	0.21*** (0.016)	1.23 [1.20, 1.27]
AIC/BIC	1538.33/1541.22		17965.32/17970.42		425.68/427.32		6896.97/6901.11	
*c*-index [95% CI]/*R*^*2*^ (XO)	0.70 [0.66, 0.75]/.09		0.65 [0.63, 0.66]/.20		0.75 [0.67, 0.82]/.17		0.67 [0.64, 0.69]/.34	
DRAOR acute	0.297*** (0.036)	1.35 [1.26, 1.44]	0.22*** (0.011)	1.25 [1.22, 1.27]	0.38*** (0.059)	1.47 [1.31, 1.65]	0.27*** (0.017)	1.31 [1.26, 1.35]
AIC/BIC	1527.62/1530.50		17893.08/17898.18		414.86/416.50		6836.60/6840.74	
*c*-index [95% CI]/*R*^*2*^ (XO)	0.70 [0.65, 0.74]/.23		0.65 [0.64, 0.67]/.28		0.78 [0.70, 0.85]/.42		0.70 [0.67, 0.72]/.42	
DRAOR protect	−0.23*** (0.033)	0.80 [0.75, 0.85]	−0.198*** (0.011)	0.82 [0.80, 0.84]	−0.35*** (0.061)	0.70 [0.62, 0.79]	−0.23*** (0.017)	0.80 [0.77, 0.82]
AIC/BIC	1547.26/1550.14		17947.30/17952.40		419.91/421.55		6894.23/6898.37	
*c*-index [95% CI]/*R*^*2*^ (XO)	0.66 [0.61, 0.71]/.10		0.65 [0.63, 0.67]/.21		0.75 [0.66, 0.83]/.42		0.68 [0.65, 0.70]/.32	
RoC*RoI (static risk)	3.298*** (0.37)	27.1 [13.0, 56.4]	2.81*** (0.17)	16.6 [11.9, 23.0]	3.798*** (0.75)	44.6 [10.2, 195.5]	3.88*** (0.31)	48.3 [26.5, 88.0]
DRAOR stable	0.022 (0.043)	1.02 [0.94, 1.11]	0.021 (0.014)	1.02 [0.99, 1.05]	−0.037 (0.087)	0.96 [0.81, 1.14]	0.021 (0.023)	1.02 [0.98, 1.07]
DRAOR acute	0.15*** (0.043)	1.16 [1.07, 1.26]	0.13*** (0.014)	1.13 [1.10, 1.17]	0.16* (0.079)	1.18 [1.01, 1.37]	0.16*** (0.021)	1.18 [1.13, 1.23]
DRAOR protect	−0.098* (0.044)	0.91 [0.83, 0.99]	−0.075*** (0.015)	0.93 [0.90, 0.96]	−0.18* (0.086)	0.84 [0.71, 0.99]	−0.061* (0.024)	0.94 [0.90, 0.99]
AIC/BIC	1434.13/1445.66		17471.03/17491.42		382.94/389.49		6601.32/6617.89	
*c*-index [95% CI]/*R*^*2*^ (XO)	0.81 [0.77, 0.84]/.61		0.73 [0.71, 0.74]/.55		0.87 [0.82, 0.92]/.76		0.77 [0.75, 0.79]/.74	

*Note*. Sexual Index *n* = 562 people, *n* = 597 release events, *n* = 17,175 weeks of follow-up for all recidivism outcomes, *n* all recidivism events = 132, *n* = 18,182 weeks of follow-up for criminal recidivism outcomes, *n* criminal recidivism events = 38. Nonsexual Index *n* = 2854 people, *n* = 3051 release events, *n* = 74,770 weeks of follow-up for all recidivism outcomes, *n* all recidivism events = 1,210, *n* = 80,985 weeks of follow-up for criminal recidivism outcomes, *n* criminal recidivism events = 466. BIC was calculated using the number of recidivism events as the sample size (see Volinsky & Raftery, 2000).

RoC*RoI = Risk of Reconviction*Risk of Re-imprisonment (Bakker et al., 1999); DRAOR = Dynamic Risk Assessment for Offender Re-entry (Serin, 2007); *c*-index = concordance index; *c*-index = concordance index; CI = confidence interval; AIC = Akaike Information Criteria; BIC = Bayesian Information Criteria; HR = hazard ratio; *R*^*2*^ (XO) = Xu & O’Quigley’s (1999) *R*^*2*^.

*p ≤ .05. **p ≤ .01. ***p ≤ .001.

Testing multivariate models in which we entered all three DRAOR scales simultaneously after first accounting for static risk (i.e., RoC*RoI scores), the pattern of results suggested that DRAOR Acute and DRAOR Protect scale scores added incrementally beyond static risk scores, whereas predictive discrimination effects associated with DRAOR Stable scores were no longer statistically significant. Finally, we repeated the multivariate models displayed in Table 3 for people convicted of sexual offenses subdividing the sample further based on whether the convicted offenses involved a victim under the age of 16 or aged 16 and older (see Table 4). Model results using the subsample with older victims was consistent with results from Table 3 such that DRAOR Acute and Protect subscale scores showed incremental validity over static risk scores. By contrast, only DRAOR Acute showed incremental validity among the subsample with victims under the age of 16.

**Table 4.** Cox Regression with Time-Varying Predictor Models Predicting Time to Recidivism Among People Incarcerated for a Sexual Offense with a Victim Younger than 16 or Victim 16 and Older Using Static Risk (RoC*RoI) and DRAOR Subscale Scores.

Parameter	Sexual Index Offense Victim Under 16 Years Old (*n* = 361)		Sexual Index Offense Victim 16 Years or Older (*n* = 207)	
	*B* (*SE*)	HR [95% CI]	*B* (*SE*)	HR [95% CI]
RoC*RoI (static risk)	3.22*** (0.53)	25.1 [8.84, 71.1]	3.39*** (0.64)	29.6 [8.52, 103.0]
DRAOR stable	0.073 (0.0598)	1.08 [0.96, 1.21]	−0.035 (0.063)	0.97 [0.85, 1.09]
DRAOR acute	0.17** (0.059)	1.18 [1.05, 1.33]	0.13* (0.065)	1.14 [1.00, 1.29]
DRAOR protect	−0.062 (0.064)	0.94 [0.83, 1.07]	−0.13* (0.064)	0.88 [0.77, 0.99]
AIC/BIC	674.01/682.88			539.69/548.07
*c*-index [95% CI]/*R*^*2*^ (XO)	0.81 [0.76, 0.86]/.56			0.79 [0.74, 0.84]/.62

*Note*. Sexual Index Victim Under 16 *n* = 344 people, *n* = 361 release events, *n* = 9870 person-period weeks of follow-up, *n* recidivism events = 68. Sexual Index Victim 16 or Older *n* = 191 people, *n* = 207 release events, *n* = 5057 person-period weeks of follow-up, *n* recidivism events = 60. BIC was calculated using the number of events as the sample size (see Volinsky & Raftery, 2000).

RoC*RoI = Risk of Reconviction*Risk of Re-imprisonment (Bakker et al., 1999); DRAOR = Dynamic Risk Assessment for Offender Re-entry (Serin, 2007); *c*-index = concordance index; CI = confidence interval; AIC = Akaike Information Criteria; BIC = Bayesian Information Criteria; HR = hazard ratio; *R*^*2*^ (XO) = Xu & O’Quigley’s (1999) *R*^*2*^.

*p ≤ .05. **p ≤ .01. ***p ≤ .001.

## Discussion

The goal of this study was to evaluate whether items and scales within a case management tool designed to predict general recidivism outcomes among the general population of people on community supervision (i.e., DRAOR) may be validly used with people on parole for convictions for sexual offenses. Because we were unable to directly compare predictive discrimination validity of DRAOR in our sample to a tool designed specifically for case management with people convicted of sexual offenses (e.g., Acute-2007), we evaluated whether the association between DRAOR scores and general recidivism may be different among subgroups. Further, because tools designed for rapid reassessment within community corrections (including DRAOR and Acute-2007) are focused on the faster-changing, acute dynamic risk factors that represent core areas of concern within case management, we evaluated whether frequently reassessed DRAOR scores (every 1–2 weeks) demonstrated predictive discrimination of imminent (prior to the next assessment) general recidivism (with and without breaches).

Overall, results demonstrated each independent DRAOR item and scale domain scores (Stable, Acute, and Protect) showed predictive discrimination validity among the subgroup of people convicted of sexual crime, and, compared to the sample of people convicted for nonsexual crimes, discrimination effects were not reduced, but were generally slightly stronger. Effects were generally medium-to-large. Next, DRAOR Acute emerged as the dynamic scale with the most consistent incremental validity beyond static risk scores, with DRAOR Protect additionally demonstrating incremental validity among people convicted of sexual crimes with victims aged 16 or older, suggesting results among this subgroup were more closely similar to the results observed among people convicted of nonsexual crime compared to those with victims younger than 16. Because DRAOR Protect factors are conceptualized as strengths rather than risk factors, it is not clear from this finding that people with underage victims have specialized risk profiles; however, the definition of strengths or their role for risk of recidivism may differ in this subgroup, notwithstanding this group’s relatively low rate of general recidivism outcomes.

Effect sizes from models using DRAOR scales were not substantively different from prediction effect sizes others have reported using tools specifically designed to assess risk for sexual recidivism. Hanson et al. (2021) reported a model concordance of .74 [95% CI = .70, .77] when predicting any recidivism (including breaches) using time-dependent SOTIPS scores across a maximum 4.2-year follow-up, and we observed a model concordance of .70 [95% CI = .66, .75] using time-dependent DRAOR Stable scores across a maximum 1-year follow-up. Similarly, time-dependent Acute-2007 scores discriminated general recidivism across an average 7.2-year follow-up with *c*-index = .64 [95% CI = .58, .69] (Babchishin & Hanson, 2020) and the equivalent DRAOR Acute effect in this study was *c*-index = .70 [95% CI = .65, .74].

### Implications for Conceptualizing Recidivism Risk

Although our analyses were novel by using reassessments of a general risk tool to examine short-term general recidivism among people convicted of sexual crime, it is, more broadly, unsurprising that general risk factors discriminate general recidivism in this cohort. Yet, this consistent finding raises questions about how to best conceptualize general recidivism factors among people convicted of sexual crime. One approach is to consider general risk factors as one of several dimensions of risk. In this conceptualization, there are different propensities for different types of behaviors and the specific combination of propensities determines the recidivism outcome. In other words, general risk factors drive the likelihood of any criminal behavior while sex-specific risk factors drive the likelihood of problematic sexual behaviors, and both “ingredients” are required for sexual recidivism to occur. For example, although the subset of items on Static-99R (Hanson & Thornton, 2000; Helmus et al., 2012) and Stable-2007 (Hanson et al., 2007) that assess general criminality predict all definitions of recidivism (i.e., sexual only, nonsexual violent only, or any recidivism; Babchishin et al., 2016; Brouillette-Alarie et al., 2016, 2018; Etzler et al., 2020; Jung et al., 2018), items that describe sexual criminal history, victims of sexual crimes, deviant sexual interests, and emotional identification with children demonstrated relatively strong specificity by (1) predicting only sexual recidivism and/or (2) showing an inverse relationship with nonsexual violent and any recidivism outcomes after statistically controlling for items that assess general criminality (Babchishin et al., 2016; Brouillette-Alarie et al., 2016, 2018; Etzler et al., 2020). These findings suggest distinct dimensions of propensity. In this conceptualization, the future of risk assessment, as Brouillette-Alarie et al. (2016, 2018) have argued, will involve using factor analysis to aggregate risk items into domains that have conceptual value and can be appropriately weighted. The ideal dataset for this approach would require a very large set of risk items that assess interrelated subcomponents within the same risk domains; risk tools are generally not constructed this way.

Further, when examining dynamic risk tools, existing factor analyses do not always provide conceptually clear evidence of general and sex-specific dimensions. For example, Babchishin and Hanson (2020) reported *sexual preoccupation* loaded alongside all other Acute-2007 items onto one latent factor, suggesting a singular construct containing various manifestations of instability and poor self- and goal-management. For Stable-2007, Etzler et al. (2020) factor analysis loaded the item *hostility toward women* onto a latent construct alongside items assessing general antisociality rather than with the two Stable-2007 items that assess positive attitudes toward sex with underage victims. This finding is problematic from a dimensional approach while also likely indicating the presence of two subgroups within Etzler et al. (2020) sample, i.e., people with adult victims who are at greater risk for general recidivism and people with child victims who are at greater risk for sexual recidivism. Yet, there is also a psychological, experiential element underlying this finding, as people with attitudes that support one criminal behavior may experience negative attitudes (and even disgust) toward another criminal behavior (e.g., Ricciardelli & Moir, 2013; Trammel & Chenault, 2009).

Thus, rather than a dimensional or “ingredient” approach, a different conceptualization of general risk factors in this cohort might focus on item-outcome concordance or the “nesting” of specific risk factors and criminal behaviors into broader risk factors and criminal behaviors. Consider, for example, the sex-specific risk factors *sexual preoccupation* (i.e., thinking about and/or engaging in sexual behavior at a higher frequency than average and to the degree of disrupted life functioning) and *using sex to cope with life difficulties*. These two factors are problems with self-regulation (a general risk factor) within the sexual domain. Other sex-specific factors are behavioral markers (or close-proxy analogues) of positive attitudes toward specific sexually violating behaviors (e.g., deviant sexual interests, hostility toward women, and emotional congruence with children), i.e., specific types of offense-supportive attitudes. Thus, these sex recidivism-specific risk domains are conceptually nested within general risk domains because they more narrowly assess how risk manifests in a specific area of life functioning.

Empirically, greater predictor-outcome correspondence is related to stronger associations. Beliefs about behaviors are more strongly associated with their specific behavior targets compared to similar but more adjacent behaviors (see Ajzen & Fishbein, 1977); for example, beliefs that it is sometimes necessary to resort to physical violence should predict assault better than drug trafficking or refugee smuggling. As such, beliefs about one criminal behavior may be inversely or unrelated to beliefs about another, even within the same broad type of criminal behavior (e.g., attitudes that support recreational MDMA use may be unrelated to heroin use, or attitudes that support sexual activity with an underage victim may be inversely related to attitudes about sexually assaulting adult women; Blanchard et al., 2001; Feelgood et al., 2005).

In this conceptualization of general risk factors, DRAOR’s general risk items and domains were associated with general recidivism outcomes in the present study because they showed item-outcome congruence. In other words, items that assess general self-regulation problems and antisocial attitudes are conceptually similar to the self-regulation breakdowns and cognitive drivers that occurred at the time of nonsexual general recidivism events. If so, had the focus of this study been on predicting a more specific behavior with greater specificity (e.g., heroin use), the assessment strategy should have assessed self-regulation and attitude patterns related specifically to heroin use. In this conceptualization, the future of risk assessment may require introducing greater conceptual specificity into assessments to better reflect how general risk factors can manifest toward anticipated outcomes in either congruent or contradictory ways, depending on the degree of specificity the assessment uses for individuals’ beliefs or capabilities.

### Implications for Practice

The current findings suggest DRAOR Acute items represent useful case management concerns that supervision officers should assess and actively manage when providing services to people convicted of sexual crime in the community. Because general (nonsexual) recidivism is the most likely recidivism outcome, especially in the first year after release from incarceration, case managers should not discount signs of general poor self-regulation or instability among clients convicted of sexual crime, even if these problems manifest in ways that seem (and may be) unrelated to the client’s risk for sexual recidivism. Within their scope of practice, supervision officers should address any dysregulated behaviors or procriminal beliefs (whether related to nonsexual or sexual functioning) as they monitor change in dynamic risk factors, challenge risk behaviors, and encourage clients to both solidify prior self-management gains and further embed risk-reducing activities into their lives. General risk factors may be more an intervention opportunity than a potential distraction from sex-specific risk factors. Marshall et al. (1999) argued that problems related to sexual deviance are nested within problems within broader (and not necessarily sexual) areas of life functioning, suggesting that clinicians may be warranted focusing rehabilitation efforts at the level of the general risk domains. If all dysregulated and procriminal problem behaviors are interconnected, there are likely numerous clinical “entry points” to address this nexus of problematic behaviors as well as cross-domain “ripple effects” that can arise from self-management skill-building within any single life domain.

DRAOR Protect factors discriminated general recidivism outcomes among the relatively younger, higher-risk cohorts (people convicted of nonsexual crimes or sexual crimes with adult victims) but not among people convicted for sexual crimes against children. On the surface, this may suggest that case managers should only focus on prosocial strengths among the former two subgroups. However, the lower static risk among people convicted for sexual crimes against children suggests this subgroup is generally and relatively more stable in their life functioning. As such, when strengths are present in these clients, case managers should focus on clients maintaining those strengths against any potential threats to their continued prosocial functioning. For example, people convicted of sexual crime may experience difficulty obtaining or maintaining social support due to stigma (Tewksbury, 2012) which may also be due to their own feelings of shame and guilt (Meloy, 2006). When such problems may lead to the relationship or lifestyle instabilities assessed on DRAOR Acute, lack of prosocial support may indirectly lead to increases in acute dynamic risk. As such, case managers should always attend to prosocial connectedness and engagement.

### Strengths and Limitations

Our conclusions about the utility of DRAOR among people convicted of sexual crime is limited by the relatively brief follow-up period. However, case managers completed assessments with enough frequency to estimate the association between DRAOR scores and imminent, short-term outcomes. Future research would benefit from conducting similar analyses with a longer follow-up period and by employing direct comparisons of DRAOR with tools specifically designed for predicting sexual recidivism, such as Stable-2007 and Acute-2007.

Our dataset did not contain full criminal histories, so we assigned subgroups by current offense. Yet, as we observed for our sample (*n* = 562), people convicted of sexual crime are more likely to recidivate with nonsexual (*n* = 118 recidivism events) than sexual crime (*n* = 6 recidivism events), meaning subsequent releases will list nonsexual index offenses. If many with nonsexual index convictions in this study (*n* = 2854) had prior convictions for sexual crime, our group comparisons would attenuate any true group differences. We were unable to find New Zealand data that specifies the percentage of individuals with nonsexual convictions who also have histories of sexual crime. However, recidivism data suggest the percentage would be small. For example, if all 118 people in this study with nonsexual recidivism during parole were re-released as part of a new similar study, this group would comprise 4.1% of the comparison group (i.e., 118/2854). Similarly, a New Zealand study of 1100 people convicted of sexual crimes released over 3 years in 2001–2003 showed 183 were reimprisoned for nonsexual convictions across 5 years follow-up (Nadesu, 2011). If all 183 people were released on parole during the timeframe of the present study, they would have represented a minority (6.4%) of our comparison group with nonsexual index offenses. Further, our victim type subsample was based on the legal definition of age of consent (i.e., age 16) rather than whether younger victims displayed secondary sexual characteristics. This made this subgroup heterogeneous in likely underlying attraction to children.

Because this was a field study using data drawn directly from a correctional agency’s database, interrater reliability of DRAOR scores is fully unknown. Also, DRAOR assessments are completed by a finite number of supervision officers, but we did not have indicators identifying which raters scored which assessments across different clients. Despite these limitations, results in this study are likely generalizable to other corrections settings similar to New Zealand Department of Corrections (e.g., in terms of training, staff engagement, quality assurance mechanisms, etc.).
